# Metabolic health and adiposity transitions and risks of type 2 diabetes and cardiovascular diseases: a systematic review and meta-analysis

**DOI:** 10.1186/s13098-023-01025-w

**Published:** 2023-03-28

**Authors:** Xuhui Zhang, Jinghan Zhu, Jean H. Kim, Timothy S. Sumerlin, Qi Feng, Jiazhou Yu

**Affiliations:** 1 Hangzhou Center for Disease Control and Prevention, Hangzhou, China; 2 The Second School of Clinical Medicine, Southern Medical University, Guangzhou, China; 3 Jockey Club School of Public Health and Primary Care, The Chinese University of Hong Kong, Sha Tin, Hong Kong; 4 Nuffield Department of Population Health, University of Oxford, Oxford, UK

**Keywords:** Obesity, Metabolic health, Transition, Type 2 diabetes, Cardiovascular disease, Meta-analysis

## Abstract

**Background:**

Metabolic health status and levels of adiposity are prone to change over time. Mixed results have been reported regarding the extent by which changes in metabolic health and weight affect cardiometabolic risks. This systematic review and meta-analysis aims to examine the association between transitions in metabolic health and adiposity status on risk of incident type 2 diabetes (T2DM) and cardiovascular disease (CVD) events.

**Methods:**

A systematic literature search was conducted on MEDLINE and EMBASE through August 2022 for prospective cohort studies examining transitions in metabolic health and adiposity status and risk of incident T2DM and CVDs without restrictions on language or publication status. Meta-analysis was performed to summarize hazard ratios for T2DM and composite CVD events separately using random-effects model.

**Results:**

A total of 17 studies were included. Compared to stable metabolically healthy status, transition to metabolically unhealthy status significantly increased the risk of incident T2DM and composite CVD events among individuals with normal weight and individuals with overweight/obesity. Compared to stable metabolically unhealthy status, transition to metabolically healthy status significantly lowered the risk among individuals with normal weight and individuals with overweight/obesity. When metabolic health status remained unchanged, progression from normal weight to overweight/obesity significantly increased risk of CVDs but not risk of T2DM.

**Conclusion:**

The impact of change in metabolic health on the risks of T2DM and CVD is more prominent than that of change to body mass index category. Obesity treatment should consider prioritizing improvement in metabolic health parameters over focusing on the extent of weight loss only.

**Supplementary Information:**

The online version contains supplementary material available at 10.1186/s13098-023-01025-w.

## Introduction

Obesity has become a worldwide epidemic which poses substantial public health and societal costs. The links between obesity and chronic diseases, including cardiovascular diseases (CVDs) and type 2 diabetes mellitus (T2DM) are well-established, and such effects are closely related to metabolic risk factors [1]. However, obesity and metabolic disorders do not always exist concurrently; for example, there is a subset of individuals with overweight/obesity that does not display metabolic abnormalities. This forms the concept of metabolic health and adiposity status, which includes a spectrum of phenotypes: metabolically healthy normal weight (MH-NW), metabolically unhealthy normal weight (MU-NW), metabolically healthy overweight/obesity (MH-O), and metabolically unhealthy overweight/obesity (MU-O).

Currently, there is no consensus on the definition of metabolic health and adiposity phenotypes. The classification criteria and specific cut-off for each parameter varied considerably across the literature [2]. Most studies defined MH-O as overweight/obesity in the absence of a metabolic disorder, such as metabolic syndrome, hypertension, abnormal glycemic traits, or dyslipidemia [3–6]. Individuals with obesity/overweight who are metabolically healthy have demonstrated increased cardiometabolic risk compared with their normal-weight counterparts [3, 7]. Some studies concluded that metabolic health plays a more important role than adiposity in predicting risk of long-term health outcomes [3–5], while some suggested otherwise [8]. Recently, it has been increasingly recognized that metabolic health and adiposity status is prone to change in nature. Such transition may alter future risk of long-term health outcomes. Prospective studies have reported that 42–85% of the MH-O individuals progress to MU-O over 7.8–20 years of follow-up [8–13]. The vast majority of studies have examined metabolic health and adiposity status phenotype at one time point under the assumption that the status remains unchanged. Despite the high likelihood of phenotypes changing over time for many adults, there is limited understanding on the health effects of metabolic health and adiposity changes. A recent meta-analysis of seven studies on metabolically healthy individuals concluded that individuals who transition to overweight/obesity status are at an elevated risk of CVDs compared to those with stable overweight/obesity status, but the analysis only considered transition from MH-O to MU-O [14]. The findings remain inconsistent with regard to the effects of positive metabolic health and adiposity transitions (metabolic health improvement and/or weight loss) and negative transitions (metabolic health deterioration and/or weight gain) on cardiometabolic risks, which is imperative for understanding potential pathways of diseases and devising individualized prevention strategies. Hence, we conducted a systematic review and meta-analysis to examine the associations between transition of metabolic health and adiposity status and risks of incident T2DM and CVD events.

## Methods

This systematic review was registered on PROSPERO (CRD42022350633) and reported according to the Preferred Reporting Items for Systematic Review and Meta-Analysis (PRISMA) [15] and Meta-analysis of Observational Studies in Epidemiology (MOOSE) [16].

### Data sources and search

We searched MEDLINE and EMBASE for relevant studies from inception through August 2022. The search strategy included three groups of keywords related to the following concept: (1) metabolic health; (2) transition; (3) type 2 diabetes, CVD events, or mortality. The search terms of these three concepts were combined using the Boolean operator “AND”. Details on search strategy are described in Additional file 1: Table S1. The reference lists of eligible studies were manually examined for additional literature. There were no restrictions on language or publication period.

### Eligibility criteria and study selection

This review included studies that investigated the association between transitions of metabolic health and adiposity phenotype (MH-NW, MU-NW, MH-O, MU-O) to another phenotype with the risks of: (1) incident type 2 diabetes, (2) incident events of composite cardiovascular disease outcomes and (3) all-cause mortality. Individuals of stable/persistent phenotype were considered the referent group in all comparisons. In this review, composite CVD events was defined as heart diseases including fatal or non-fatal coronary heart diseases, myocardial infarction, heart failure, and stroke.

Prospective cohort studies were included if they: (1) examined metabolic health and adiposity status transition over time; (2) reported relative risk for incident T2DM or of CVD events (any outcome that belongs to composite CVD events as defined above) or of all-cause mortality; (3) were published or in press with peer-reviewed journals. Studies were excluded if they: (1) were reviews, protocols, conference abstracts, or not peer-reviewed publications; (2) examined only one metabolic parameter when measuring metabolic health; (3) did not specify the direction of metabolic health and adiposity transition; (4) did not report relevant data for quantitative analysis. For duplicate reports from the same cohort, we only included the report with the largest number of cases.

The study selection followed a two-step procedure. First, the title and abstract of all electronically and manually identified records were screened to identify potentially eligible studies. Second, full texts were reviewed to determine eligibility. Two authors (XZ and JZ) independently performed study selection. All disagreements were resolved by discussion with a third reviewer until consensus was reached.

### Data extraction and quality assessment

Data were extracted using a designed form, which collects the following information: (1) study characteristics: first author, year, study country, study period, sample size; (2) participants characteristics: age, sex, ethnicity; (3) methodological characteristics: exposure definitions, ascertainment of outcome, follow-up period; (4) effect estimates for the association; and (5) other information for study quality assessment.

Newcastle–Ottawa Scale for cohort studies was used to assess the methodological quality of eligible studies. The scale covered three domains: selection of participants, comparability of study groups, and ascertainment of outcome [17]. As the representativeness of exposed cohort was not relevant given the research question of this review, this point was removed in quality assessment. A score system (score range 0–8, higher score indicating higher quality and lower risk of bias) was used to present the result of quality assessment. In this review, the quality of cohort studies was classified into high (scored 7–8), moderate (scored 4–6), and low (scored 3 or less). Two authors (XZ and JZ) independently performed data extraction and quality assessment. Any discrepancy in quality level was solved by discussion until consensus was reached.

### Data synthesis and analysis

There has been no consensus on the definition of metabolic health and adiposity phenotype. Most studies used a combination of three to five components to define metabolic health, for example, blood pressure, fasting glucose, HDL-cholesterol, triglycerides, and waist circumference. Definition of overweight/obesity also varied by study. For studies that reported data separately for normal weight, overweight, and obesity groups, data for overweight and obesity groups were included separately in analysis.

**Fig. 1:**
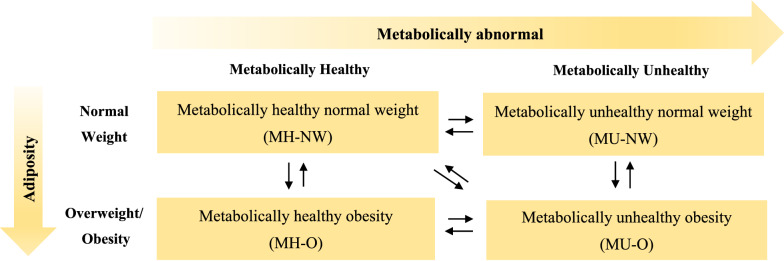
Classification of metabolic health and adiposity status and transitions

Effect measures comparing the group with transitional metabolic health and adiposity status to the group with stable status was synthesized to present the association of interest. Specifically, associations of ten transitional groups were extracted separately for T2DM and CVDs: (1) from metabolically healthy to unhealthy when adiposity status remains unchanged: MH-NW to MU-NW and MH-O to MU-O; (2) from metabolically unhealthy to healthy when adiposity status remains unchanged: MU-NW to MH-NW and MU-O to MH-O; (3) from normal weight to overweight/obesity when metabolic health remains unchanged: MH-NW to MH-O and MU-NW to MU-O; (4) from overweight/obesity to normal weight when metabolic health remains unchanged: MH-O to MH-NW and MU-O to MU-NW; (5) dual transition: MH-NW to MU-O and MU-O to MH-NW, representing transitions between the healthiest and the least healthy of the four phenotypes (Fig. 1). Hazard ratio (HR) with 95% confidence intervals (CIs) was the most used measure of effect in the original studies and was therefore used in this meta-analysis. Odds ratios (ORs), were transformed into risk ratios (RRs) using the following formula: RR = OR/[(1-P_0_) + (P_0_*OR)], where P_0_ is the risk of an event in the non-exposed group [18]. The transformed RRs and those extracted from some original studies were converted into HR using the formula: RR = (1-e^HR ln(1−r)^)/r,, where r is the rate of outcome in the reference group [19].

Effect estimates were synthesized across included studies by meta-analysis. Heterogeneity across studies was assessed by Cochran’s Q test and I^2^, with p < 0.05 and/or I^2^ > 50% indicating significant heterogeneity [20, 21]. Subgroup analyses were performed to detect source of heterogeneity, if any, as well as potential effect modification, but only when the number of included studies was more than five. Subgroup analyses were prespecified according to study setting (North America, Europe, Asia), mean age (< 50, ≥ 50), and ethnicity (Caucasian predominant, Asian, Persian) of participants, cut-off for overweight/obesity [body mass index (BMI) = 24, 25, 30], number of components to define metabolic health status (3, 4, 5 components), follow-up time (< 5, ≥ 5 years for T2DM; < 10, ≥ 10 years for CVDs), evaluation of transition in metabolic health and weight (prior to follow-up, during early phase of follow-up, throughout follow-up), confounders (age, sex, lifestyle factors; age, sex, lifestyle factors, clinical parameters), analysis excluding cases occurred during early follow-up (yes, no), and quality of study (low, moderate, high). Potential publication bias was assessed by visual inspection of funnel plots as well as the Egger’s test when the number of included studies is more than ten [22]. A p-value < 0.10 in Egger’s test indicates presence of publication bias. The trim-and-fill method was used to adjust for any significant publication bias detected [23]. For studies that reported data separately for normal weight, overweight, and obesity groups, we conducted sensitivity analysis where estimates for overweight and obesity groups were pooled using fixed-effect method before inclusion in the analysis.

Given the heterogeneity in study population characteristics and definition of metabolic health and adiposity phenotype, random-effects model was used for all meta-analyses. All quantitative analyses were conducted using Stata 14.0 (Stata Corp LP, College Station, TX, USA).

### Grading of evidence

The Grading of Recommendations, Assessment, Development, and Evaluation (GRADE) approach was used to rate the certainty of evidence of each association as very low, low, moderate, and high [24]. According to the guideline, rating of observational studies started as “low” certainty and was rated down based on risk of bias, inconsistency, indirectness, imprecision, publication bias, and was rated up if there was large effect size, a dose–response relationship, or attenuation by plausible confounders.

## Results

### Summary of study selection

**Fig. 2:**
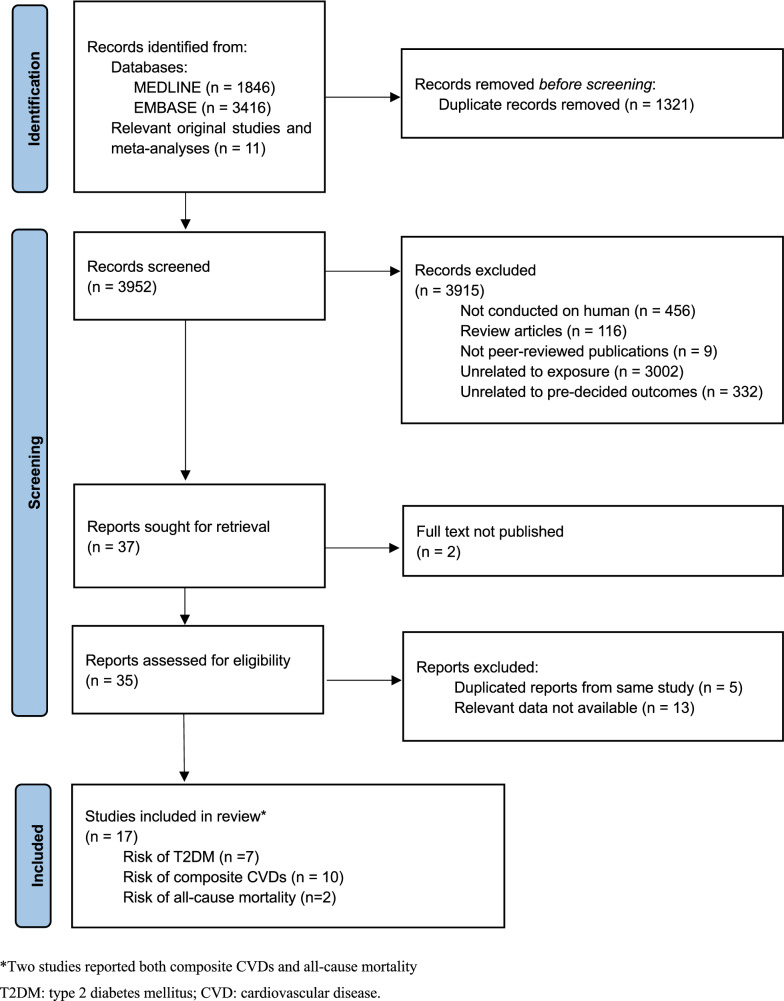
Flow chart for study selection

A total of 5,262 records were identified from the literature search, and an additional 11 records were obtained by manual examination of the reference lists of related studies. After removing duplicates and screening titles and abstracts, 37 reports were sought for retrieval. Two reports without published full-text articles were excluded. After assessment of full texts, five duplicate reports and 13 studies without relevant data were excluded. Finally, 17 studies were considered eligible to be included in this systematic review. Among the eligible studies, seven reported the risk of incident T2DM [25–31], ten studies reported the risk of incident composite CVD events [9, 32–40], and two studies reported risk of all-cause mortality [32, 39]. The process of study selection is demonstrated in Fig. 2.

### Characteristics of included studies

The characteristics of included studies are described in Table 1 and Additional file 1: Table S2. The majority of studies were conducted in Asia (seven in China, four in South Korea, one in Japan, one in Iran), two in the United States, and two in Europe. The mean sample size was 486,419, with a range from 2692 to 7,148,763. The mean age ranged from 42.8 to 61.1 years. One study included only females [9] and the rest of the studies were conducted in both sexes, one of which reported data separately for males and females [35]. The median follow-up ranged from 3.7 to 24.0 years. Assessment of metabolic health and obesity varied across studies. Six studies considered five components for determination of metabolic health, including blood pressure, plasma glucose, HDL-cholesterol, triacylglycerols, and waist circumference [27, 28, 30, 32, 34, 35, 37]; eight studies considered four components, including blood pressure, plasma glucose, HDL-cholesterol, and triacylglycerols [25, 26, 29, 31, 32, 38–40]; one study examined four components, including blood pressure, plasma glucose, triacylglycerols, and waist circumference [33]; the remaining two studies examined three components [9, 36]. Overweight/obesity was defined as BMI ≥ 24 kg/m^2^ in five studies [25, 28, 30, 31, 33], ≥ 30 kg/m^2^ in two studies [29, 39], and ≥ 25 kg/m^2^ in the remaining studies. Five studies examined transition of metabolic health and adiposity status prior to initiation of follow-up [9, 26, 32, 37, 40], five studies examined transition during the early phase of follow-up (mainly within the first 3–8 years) [27, 29, 33, 34, 36], and seven studies examined transition over the entire follow-up period from baseline to the end of follow-up [25, 28, 30, 31, 35, 38, 39].

**Table 1:** Basic characteristics of the included studies (n = 17)

Study ID	Country	Sample size	Mean age	Male %	Median FU year	Outcome assessed	Metabolic health components				
							Blood pressure	Plasma glucose	HDL-C	Triacygly-cerols	WC
Feng 2020	China	49,702	66 *	43.7	4.0	T2DM	X	X	X	X	
Heianza 2014	Japan	27,478	47.3	64.5	6.0	T2DM	X	X	X	X	
Lee 2015	South Korea	2692	61.7	61.6	8.0	T2DM	X	X	X	X	X
Min 2021	China	4604	54.0	33.8	3.0	T2DM	X	X	X	X	X
Navarro-González 2016	Europe	4340	54.6	50.3	9.0 (male) 9.2 (female)	T2DM	X	X	X	X	
Song 2022	China	14,894	57.5	39.6	10.1	T2DM	X	X	X	X	X
Wang 2018	China	11,865	49.9	38.0	6.0	T2DM	X	X	X	X	
Cho 2019	South Korea	362,863	58.9	53.7	2.0	Composite CVD events: admissions for MI and stroke	X	X	X	X	
Eckel 2018	US	90,257	46.3	0	24.0	Composite CVD events: MI, stroke	X	X	X		
Gao 2020	China	458,246	50.9	40.8	10.0	Composite CVD events: vascular death, MI, stroke	X	X		X	X
Guo 2021	China	7472	53.5	46.6	4.7	Composite CVD events: CHD, stroke	X	X	X	X	X
Hosseinpanah 2020	Iran	6758	42.8	41.6	15.9	Composite CVD events: CHD, stroke, CVD death	X	X	X	X	X
Lee 2022	South Korea	6665	51.7	47.3	17.4	Composite CVD events: MI, congestive heart failure, CAD, cerebrovascular disease, peripheral artery disease	X	X		X	
Lee 2020	South Korea	7,148,763	48.7	56.5	3.7	Composite CVD events: hospitalization for heart failure	X	X	X	X	X
Lin 2020	China	6220	53.9	37.1	4.4	Composite CVD events: MI, stroke, hospitalization or treatment of heart failure	X	X	X	X	
Mongraw-Chaffin 2018	US	5005	61.1	51.8	12.2	Composite CVD events: CHD, stroke, heart failure	X	X	X	X	
Mørkedal 2014	Norway	61,299	48.8	46.1	12.2	Composite CVD events: MI	X	X	X	X	

FU: follow-up; HDL-C: high-density lipoprotein cholesterol; WC: waist circumference; T2DM: type 2 diabetes mellitus; CVD: cardiovascular disease; MI: myocardial infarction; CHD: coronary heart disease; CAD: coronary artery disease; *median age

### Quality assessment

The results of study quality assessment are displayed in Additional file 1: Table S3. The included studies received stars ranging from five to eight according to the Ottawa-Newcastle scale for cohort studies. Twelve studies were rated as high quality [9, 25–27, 29, 31–35, 37, 40] while five were rated as moderate quality [28, 30, 36, 38, 39]. In studies of moderate quality, the common bias was from inadequate follow-up of cohorts.

### Meta-analysis of risk for T2DM

#### Transitions between metabolically healthy and unhealthy phenotypes

**Fig. 3:**
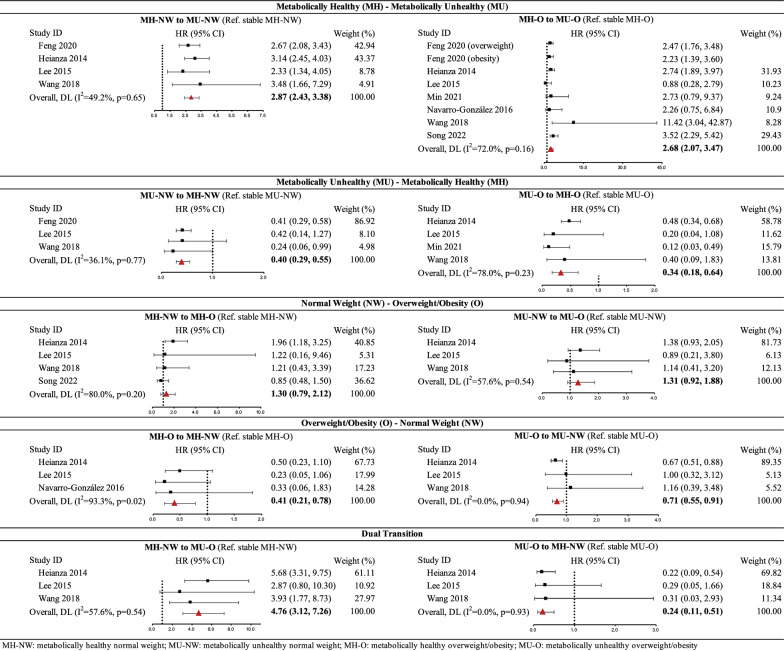
The forest plots for risk of type 2 diabetes mellitus

The results of meta-analyses on ten associations for risk of T2DM are demonstrated in Fig. 3. After combining 91,737 participants including 3205 cases from four studies [25–27, 31], transition from MH-NW to MU-NW was associated with significantly higher risk of T2DM compared to stable MH-NW status (HR 2.87, 95% CI 2.43–3.38, I^2^ = 49.2%). The summary estimate of 115,575 participants including 12,758 cases from seven studies [25–31] showed that transition from MH-O to MU-O was associated with significantly higher risk of T2DM compared to stable MH-O status (HR 2.68, 95% CI 2.07–3.47, I^2^ = 72.0%). The sensitivity analysis that included pooled estimates for overweight and obesity groups showed a similar result (HR 2.77, 95% CI 2.06–3.73). Subgroup analysis showed a consistent positive association between transition from MH-O to MU-O and T2DM risk within all subgroups (HR ranging from 1.43 to 4.83), although statistical significance was not reached in some subgroups (Additional file 1: Figure S1).

Summary estimates of 64,259 participants including 1,950 cases from three studies [25, 27, 31] showed that transition from MU-NW to MH-NW was associated with significantly lower risk of T2DM compared to stable MU-NW status (HR 0.40, 95% CI 0.29–0.55, I^2^ = 36.1%). Conversely, transition from MU-O to MH-O was associated with lower risk of T2DM compared to stable MU-O status [46,639 participants including 2,420 cases from four studies [26–28, 31]; HR 0.34, 95% CI 0.18–0.64, I^2^ = 78.0%].

#### Transitions between normal weight and overweight/obesity phenotypes

The summary results showed that transition from normal weight to overweight/obesity showed a non-significant harmful effect on T2DM risk among metabolically healthy individuals [56,929 participants including 11,195 cases from four studies [26, 27, 30, 31]; HR 1.30, 95% CI 0.79–2.12, I^2^ = 80.0%] and metabolically unhealthy individuals [42,035 participants including 2,162 cases from three studies [26, 27, 31]; HR 1.31, 95% CI 0.92–1.88, I^2^ = 57.6%].

Compared to stable overweight/obesity status, transition to normal weight showed a significant protective effect on T2DM risk among both metabolically healthy individuals [34,510 participants including 1,519 cases from three studies [26, 27, 29]; HR 0.41, 95% CI 0.21, 0.78, I^2^ = 93.3%] and metabolically unhealthy individuals [42,035 participants including 2,162 cases from three studies [26, 27, 31]; HR 0.71, 95% CI 0.55–0.91, I^2^ = 0.0%].

#### Dual transitions between MH-NW and MU-O

After combining 42,035 participants including 2,162 cases from three studies [26, 27, 31], transition from MH-NW to MU-O was associated with almost fivefold higher risk of T2DM compared to stable MH-NW status (HR 4.76, 95% CI 3.12–7.26, I^2^ = 57.6%); conversely, transition from MU-O to MH-NW lowered the risk to a similar magnitude (HR 0.24, 95% CI 0.11–0.51, I^2^ = 0.0%).

### Meta-analysis of risk for composite CVDs

#### Transitions between metabolically healthy and unhealthy phenotypes

**Fig. 4:**
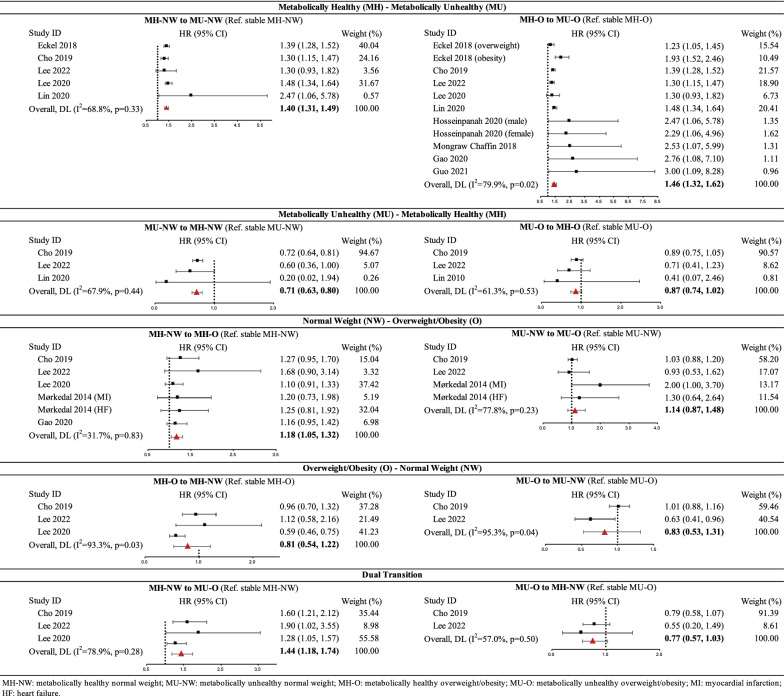
The forest plots for risk of composite cardiovascular disease events

The results of meta-analyses on ten associations for risk of incident composite CVD events are described in Fig. 4. After combining a total of 7,614,768 participants including 22,018 cases from five studies [9, 32, 36–38], transition from MH-NW to MU-NW was associated with higher risk of CVDs compared to stable MH-NW status (HR 1.40, 95% CI 1.31–1.49, I^2^ = 68.8%); no subgroup analysis was conducted to explore potential sources of heterogeneity due to the small number of studies included. After combining a total of 8,092,249 participants including 76,232 cases from nine studies [9, 32–37, 39], transition from MH-O to MU-O was associated with higher risk of CVDs compared to stable MH-O status (HR 1.46, 95% CI 1.32–1.62, I^2^ = 79.9%). The sensitivity analysis that included pooled estimates for overweight and obesity groups showed a similar result (HR 1.40, 95% CI 1.28–1.52). Although the main analysis showed significant heterogeneity between studies (p = 0.02), subgroup analyses based on the prespecified criteria showed non-significant differences (p > 0.05). The associations between transition from MH-O to MU-O and risk of composite CVD events were consistently significant within all subgroups, with HR ranging from 1.40 to 2.76 (Additional file 1: Figure S2).

Three studies [32, 36, 38] with 375,748 participants and 12,561 cases were included in analysis for transition from unhealthy to healthy metabolic status. Results showed that compared to stable unhealthy status, this transition was associated with significantly lower risk of CVDs among normal weight individuals (HR 0.71, 95% CI 0.63–0.80, I^2^ = 67.9%) but showed non-significant association with individuals with overweight/obesity (HR 0.87, 95% CI 0.74–1.02 I^2^ = 61.3%). No subgroup analysis was conducted due to the small number of studies included.

#### Transitions between normal weight and overweight/obesity phenotypes

Combining 8,037,836 participants including 69,668 cases from four studies [32, 33, 36, 40], we found that transition from MH-NW to MH-O was associated with higher risk of CVDs compared to stable MH-NW status (HR 1.18, 95% CI 1.05, 1.32, I^2^ = 31.7%). Three studies [32, 36, 40] with 430,827 participants and 14,266 cases were included in analysis for transition from MU-NW to MU-O, and summary HR for CVDs was not statistically significant (HR 1.14, 95% CI 0.87–1.48, I^2^ = 77.8%).

The summary estimates showed that compared to stable overweight/obesity status, transition to normal weight showed a non-significant protective effect for CVD risk among metabolically healthy individuals [7,518,291 participants including 14,870 cases from three studies [32, 36, 37]; HR 0.81, 95% CI 0.54–1.22, I^2^ = 93.3%] and metabolically unhealthy individuals [369,528 participants including 11,719 cases from two studies [32, 36]; HR 0.83, 95% CI 0.53–1.31 I^2^ = 95.3%].

#### Dual transitions between MH-NW and MU-O

Combining 7,518,291 participants including 14,870 cases from three studies [32, 36, 37], transition from MH-NW to MU-O was associated with significantly higher risk of CVDs compared to stable MH-NW status (HR 1.44, 95% CI 1.18–1.74, I^2^ = 78.9%). The summary HR for 369,528 participants including 11,719 cases from two studies [32, 36] showed a non-significant protective effect of transition from MU-O to MH-NW on CVD risk (HR 0.77, 95% CI 0.57–1.03, I^2^ = 57.0%).

When stratified by the original CVD outcomes as reported in the included studies instead of composite CVD events, the results were generally consistent with those reported above (Additional file 1: Figure S3).

**Fig. 5:**
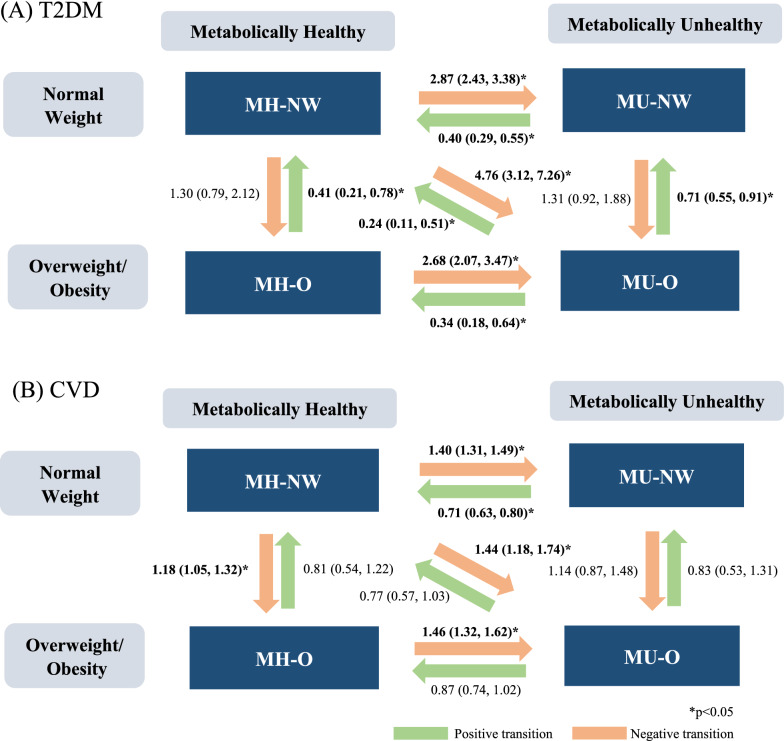
Summary hazard ratios (95% confidence interval) for **A** type 2 diabetes mellitus; **B** cardiovascular diseases

In summary, the synthesized estimates of associations between transitions in metabolic health and adiposity phenotypes and risk for T2DM and composite CVDs are illustrated in Fig. 5.

### Meta-analysis of risk for all-cause mortality

Combining 367,868 participants with 2,917 cases of two studies [32, 39], the analysis showed that transition from MH-O to MU-O did not significantly affect the risk of all-cause mortality (HR 0.98, 95% CI 0.64–1.50) (Additional file 1: Figure S3). For other transitional phenotype groups, only one study was included [32]. In this study, transition from MH-NW to MU-NW (HR 1.24, 95% CI 1.13–1.37) and transition from MU-O to MU-NW (HR 1.31, 95% CI 1.15–1.49) significantly increased the risk for all-cause mortality; the remaining groups all showed non-significant results (p > 0.05).

### Publication bias

Egger’s test was conducted for analysis of transition from MH-O to MU-O and CVD risk. The results suggested there was evidence of significant publication bias (p = 0.04). After imputing three studies using the trim-and-fill method, the adjusted funnel plot showed an overall asymmetrical pattern. The effect size of the adjusted model (HR 1.42, 95% CI 1.28–1.57) was not significantly different from the original effect size (HR 1.46, 95% CI 1.32–1.62), suggesting that the presence of publication bias did not significantly affect the validity of results. The original and adjusted funnel plots are shown in Additional file 1: Figure S4. Publication bias was not examined for other associations, as their numbers of studies included were smaller than ten.

### Grading of evidence

The certainty of evidence was rated as “moderate” for six, “low” for six, “very low” for eight of the associations (Additional file 1: Tables S5 and S6). The certainty of evidence was strongest for association between transition from MH-NW to MU-NW, MH-O to MU-O, MU-NW to MU-NW to MH-NW, MU-O to MH-O, MH-NW to MU-O, MU-O to MH-NW and risk of T2DM. These associations were rated as “moderate” after rated upward for large effect size, whereas associations rated as “very low” were rated downward largely for imprecision, inconsistency, and publication bias. In general, associations with risk of T2DM showed higher certainty of evidence than those with risk of CVD.

## Discussion

This systematic review and meta-analysis found that transitions between different metabolic health and adiposity status alter the risk of long-term health outcomes. Compared to stable metabolically healthy status, deterioration of metabolic health increased T2DM risk by approximately 2.8-fold and CVD risk by 1.4-fold, irrespective of obesity status. Compared to stable metabolically unhealthy status, improvement in metabolic health lowered the risk of T2DM by 60% and CVDs by 30% among normal weight individuals and lowered the risk of T2DM by 66% among individuals with overweight/obesity. When metabolic health status remained unchanged, progression from normal weight to overweight/obesity only significantly increased the risk of CVDs among metabolically healthy individuals and did not affect the risk of T2DM. Concurrent improvement in metabolic health and obesity status produced a synergetic fivefold protective effect on risk of T2DM.

The concept of metabolically healthy obesity has been controversial. Several meta-analyses have concluded that MH-O is not necessarily a benign condition as they report that baseline MH-O is associated with increased T2DM and CVD risks compared to baseline MH-NW [3–7]. There is evidence that only approximately half or less of MH-O individuals are able to maintain their healthy status over time [8, 11–13, 27, 32]. Our analysis further reveals that loss of metabolically healthy status generally increases the risk of T2DM and CVDs, which partially explains the association found between baseline MH-O and cardiometabolic risks, especially in studies with long follow-up.

Our results note that in general, the effect of transition between metabolically healthy and unhealthy status on T2DM and CVD risks is stronger than that of transitions between normal weight and overweight/obesity. Some researchers suggest that MH-O is an intermediate status between MH-NW and MU-O [41, 42]. It is reported that MH-O individuals are more likely to lose the metabolically healthy status than MH-NW individuals over a long follow-up period [9, 11, 13]. As obesity is closely related to metabolic disorders, it is possible that those who transitioned from MH-NW to MH-O may experience a lag in risk when they progress to metabolic abnormalities and resultant increase in cardiometabolic risk.

Past evidence has shown that unhealthy metabolic status is associated with higher cardiometabolic risk regardless of BMI status, with MU-O individuals presenting the highest risk [3, 5, 7, 43]. Our results have suggested that in general, positive transitions to metabolically healthy status and/or normal weight could help reduce cardiometabolic risks, although the associations differed between T2DM and CVDs. For T2DM, all the positive transitions (metabolically unhealthy to healthy, obesity to normal weight) showed significant protective effects, which may support the reversibility of the pathophysiological pathways of increased T2DM risk through metabolic health deterioration and/or weight gain. Positive transitions are uncommon through natural course, only observed in 9.4–16.0% of participants in the included studies. Therefore, in addition to preventing progression to unhealthy statuses, interventions for individuals who are metabolically unhealthy and/or with obesity may also be beneficial, although to a lesser extent, for reducing risk for T2DM. For CVDs, the risk did not significantly decrease after the MU-O group underwent transition to MU-NW, MH-O, or even MH-NW, which may suggest that some of the pathophysiological pathways by which CVD risk is increased through progression to MU-O cannot be reversed by metabolic or weight interventions or the benefit may require a longer time to demonstrate as compared with T2DM risk. Additionally, transition to normal weight showed similar beneficial but non-significant effects in individuals with metabolically healthy or unhealthy obesity. These results indicate that to reduce CVD risk, improvement in metabolic health may be more beneficial than weight loss; preventing progression to MU-O may be more meaningful than interventions on individuals with unhealthy statuses.

Studies show that MH-O and MU-O individuals respond differently to lifestyle interventions and weight loss, and MH-O may benefit less from obesity treatment than MU-O [44–46]. However, to date, no trials have been conducted to compare the effect of interventions on cardiometabolic risks between MH-O and MU-O groups. In obesity treatment, the MU-O group, which carries the highest risk of adverse cardiometabolic outcomes [3, 5, 7, 43], should be prioritized with focus on metabolic health parameters rather than the extent of weight loss only. MH-O could be set as an intermediate goal of treatment. Several interventions, such as change in quality of diet, adoption of a healthy eating pattern such as the Mediterranean diet, and regular exercise training may help shift MU-O to MH-O [47, 48]. For MH-O individuals, although transition to normal weight is ideal, the primary goal could be maintaining the metabolic health profile and prevent progressing into unhealthy status with a moderate level of weight loss. Aside from the group with obesity, MU-NW individuals, who are also at an increased cardiometabolic risk compared to MH-NW individuals, should not be neglected in prevention or clinical treatment. There is evidence that metabolically unhealthy status in normal-weight individuals is closely related with visceral obesity and fatty liver, and MU-NW phenotype may be characterized by impaired insulin resistance and low cardiorespiratory fitness [49]. Hence, once metabolic abnormalities are detected, it is important to determine whether the MU-NW individual is accompanied by these conditions, which requires timely intervention to prevent further progression. Additionally, lifestyle interventions to facilitate transition to MH-NW could help reduce health burden related to T2DM and CVDs. For prevention of adverse health outcomes among healthy populations, it would be strategic to regularly monitor the metabolic status on top of body weight and aim for maintaining metabolic health by adopting healthy lifestyles.

This meta-analysis, to the authors’ knowledge, is the first to systematically examine the association between transitions between various metabolic health and adiposity phenotypes and risks of incident T2DM and CVD events. In contrast to the majority of existing studies that focus on baseline status only, we consider the transient nature of metabolic health and weight. Our main findings support that metabolic health determined by a single time point may lead to underestimation when establishing risk of long-term outcomes. Implementing standardized phenotyping by metabolic and adiposity status and monitoring the status transition over time will help with risk stratification and individualized interventions in both clinical and community settings. Other strengths include an exhaustive literature search, inclusion of prospective cohort studies of large sample size and moderate to high methodological quality, and assessment of certainty of evidence.

This study has several limitations. First, the definition of metabolic health and overweight/obesity varied across studies, which may introduce heterogeneity in meta-analysis. However, we used random-effects model to minimize the effect of heterogeneity on the overall estimates. Subgroup analysis was performed to explore heterogeneity caused by this variation and showed consistent association within each group. For future research and clinical practice purposes, there is an urgent need to establish a consensus on the definition for metabolic health and adiposity status. Second, consistent with most existing studies, this review focused on four main phenotypes based on categorized metabolic health and BMI. Given the fact that changes in metabolic health parameter and weight are continuous, future research may further explore risk evaluation on a continuous scale. Third, most studies included in this review are from Asian settings, so more evidence is needed from other ethnic populations such as Caucasian and African populations. In some original studies, the transition of metabolic health and adiposity status was evaluated over the same period as the outcome [25, 28, 30, 31, 35, 39], which may lead to reverse causation. Additionally, despite the evidence of significant publication bias for analysis of transition from MH-O to MU-O and CVD risk, the validity of summary estimate was hardly affected according to the results of the trim-and-fill method. The number of studies included in meta-analyses for certain transitional phenotype groups were limited, which precludes the possibility of performing subgroup analysis and Egger’s test for publication bias. While results for these groups should be interpreted with caution, subgroup analysis for transition from MH-O to MU-O showed largely consistent results, which support the robustness of summary estimates from main analysis. With GRADE approach, the certainty of evidence generated from our analysis was rated between “very low” to “moderate”, and the uncertainty was largely contributed by the observational study design which precluded establishment of causality, imprecision in synthesized estimates, and substantial unexplained heterogeneity. Additional research will likely influence the certainty of our estimates. Lastly, although we also aimed to examine the association between phenotype transitions and all-cause mortality, only one study reported data of different associations. Future research may consider conducting meta-analysis on this outcome when more data are available.

In conclusion, transitions in metabolic health and adiposity status alter the risk of incident type 2 diabetes and cardiovascular diseases, and the impact of change in metabolic health status is more prominent than that of change in BMI category. Obesity treatment should focus more on metabolic health parameters rather than weight loss only. Future research is needed to explore the effect of stratified interventions for metabolically healthy and metabolically unhealthy obesity individuals.

## Supplementary Information

**Additional file 1. Table S1** Literature search strategy. **Table S2** Basic characteristics of the included studies (n=19). **Table S3** Results of quality assessment (n=19). **Table S4** Certainty of evidence assessed by GRADE for associations with risk of T2DM. **Table S5** Certainty of evidence assessed by GRADE for associations with risk of CVD. **Figure S1** Results of subgroup analysis for MH-O to MU-O and risk for T2DM. ** Figure S2** Results of subgroup analysis for transition from MH-O to MU-O and risk of composite CVD events. **Figure S3** The forest plots for risk of CVD as originally reported in studies and all-cause mortality. **Figure S4** Funnel plots for transition from MH-O to MU-O and risk of CVD events: (A) original funnel plot; (B) funnel plots generated from trim-and-fill method.

## Data Availability

Data sharing is not applicable to this article as no datasets were generated or analysed during the current study.

## Abbreviations

BMI: Body mass index
CAD: Coronary artery disease
CHD: Coronary heart disease
CI: Confidence interval
CVD: Cardiovascular disease
HDL-C: High-density lipoprotein cholesterol
HR: Hazard ratio
MH-NW: Metabolically healthy normal weight
MH-O: Metabolically healthy obesity
MI: Myocardial infarction
MOOSE: Meta-analysis of Observational Studies in Epidemiology
MU-NW: Metabolically unhealthy normal weight
MU-O: Metabolically unhealthy obesity
OR: Odds ratio
PRISMA: Preferred Reporting Items for Systematic Review and Meta-Analysis
RR: Risk ratio
T2DM: Type 2 diabetes mellitus
