# Supplementary material for: Metabolic health and adiposity transitions and risks of type 2 diabetes and cardiovascular diseases: a systematic review and meta-analysis
Source: Diabetol Metab Syndr. 2023 Mar 28;15:60. doi: 10.1186/s13098-023-01025-w (PMC10045173; doi:10.1186/s13098-023-01025-w)
Supplement: Supplementary file 1 — Additional file 1. Table S1 Literature search strategy. Table S2 Basic characteristics of the included studies (n=19). Table S3 Results of quality assessment (n=19). Table S4 Certainty of evidence assessed by GRADE for associations with risk of T2DM. Table S5 Certainty of evidence assessed by GRADE for associations with risk of CVD. Figure S1 Results of subgroup analysis for MH-O to MU-O and risk for T2DM. Figure S2 Results of subgroup analysis for transition from MH-O to MU-O and risk of composite CVD events. Figure S3 The forest plots for risk of CVD as originally reported in studies and all-cause mortality. Figure S4 Funnel plots for transition from MH-O to MU-O and risk of CVD events: (A) original funnel plot; (B) funnel plots generated from trim-and-fill method. [file 13098_2023_1025_MOESM1_ESM.docx]

**Additional Materials**

**Additional file 1: Table S1**. Literature search strategy

1. MEDLINE

| Search | Query |
| --- | --- |
| 1 | Search: metabolic*[Title/Abstract] |
| 2 | Search: ((((normal[Title/Abstract]) OR (healthy[Title/Abstract])) OR (unhealthy[Title/Abstract])) OR (abnormal*[Title/Abstract])) OR (benign[Title/Abstract]) |
| 3 | Search: #1 AND #2 |
| 4 | Search:  (dynamic[Title/Abstract]) AND (change[Title/Abstract]) |
| 5 | Search:  ((((transit[Title/Abstract]) OR (transition[Title/Abstract])) OR (transient[Title/Abstract])) OR (stable[Title/Abstract])) OR (over time[Title/Abstract]) |
| 6 | Search: #4 OR #5 |
| 7 | Search: #3 AND #6 |
| 8 | Search: ((((((((((cardiovascular disease[Title/Abstract]) OR (cvd[Title/Abstract])) OR (myocardial infarction[Title/Abstract])) OR (coronary heart disease[Title/Abstract])) OR (heart failure[Title/Abstract])) OR (angina[Title/Abstract])) OR (ischemia[Title/Abstract])) OR (stroke[Title/Abstract])) OR (mortality[Title/Abstract])) OR (diabetes[Title/Abstract])) NOT (type 1 diabetes[Title/Abstract]) |
| 9 | Search: #7 AND #8 |

(B) EMBASE

| Search | Query |
| --- | --- |
| 1 | metabolic*.ab,ti. |
| 2 | (normal or healthy or unhealthy or abnormal or benign).ab,ti. |
| 3 | 1 and 2 |
| 4 | dynamic.ab,ti. and change.ab,ti. |
| 5 | (transit or transition or transient or stable or over time).ab,ti. |
| 6 | 4 or 5 |
| 7 | 3 and 6 |
| 8 | (cardiovascular disease or cvd or myocardial infarction or coronary disease or heart failure or angina or ischemia or stroke or diabetes or mortality).ab,ti. |
| 9 | type 1 diabetes.ab,ti. |
| 10 | 8 not 9 |
| 11 | 7 and 10 |

**Additional file 1: Table S2.** Basic characteristics of the included studies (n=19).

| *Study ID* | *Study of*  *data source* | *Country* | *Sample size* | *Median FU year* | *No. of outcomes* | *Definition of metabolic health* | *Classification of*  *overweight/*  *obesity* | *Transitions in metabolic health and adiposity phenotype*  *(events / N)* | *Evaluation of*  *phenotype transition* |
| --- | --- | --- | --- | --- | --- | --- | --- | --- | --- |
| Feng 2020 | - | China | 49702 | 4.0 | 1043 T2DM | MH: 0-1 factors  MU: ≥2 factors  (1) systolic BP≥130 mm Hg and/or diastolic BP≥85 mm Hg or antihypertensive drug treatment;  (2) FPG ≥5.6 mmol/L (100 mg/dL); (3) drug treatment to increase HDL-C levels;  (4) TG ≥1.7 mmol/L (150 mg/dL). | BMI≥24 | MH-NW to MU-NW (NA / 2787)  MH-O to MU-O (NA / 4100) | Throughout FU |
| Heianza 2014 | - | Japan | 27478 | 6.0 | 1255 T2DM | MH: 0-1 factors  MU: ≥2 factors  (1) systolic BP≥130 mmHg and/or a diastolic BP≥85 mmHg or treatment; (2) fasting glucose 100-125 mg/dL (5.6–6.9 mmol/L); (3) HDL-C concentration: <40 mg/dL (1.03 mmol/L) in male and <50 mg/dL (1.29 mmol/L) in female;  (4) triglycerides ≥150 mg/dL (1.7 mmol/L) or treatment. | BMI≥25 | MH-NW to MU-NW (86 / 1753)  MH-O to MU-O (69 / 648)  MU-NW to MH-NW (68 / 1412)  MU-O to MH-O (49 / 547)  MH-NW to MH-O (16 / 539)  MU-NW to MU-O (35 / 247)  MH-O to MH-NW (7 / 359)  MU-O to MU-NW (21 / 157)  MH-NW to MU-O (14 / 157)  MU-O to MH-NW (4 / 114) | Prior to FU |
| Lee 2015 | CMC | South Korea | 2692 | 8.0 | 214 T2DM | MH: 0-2 factors  MU: ≥3 factors  (1) BP≥130/85 mmHg, or current use of antihypertensive medication;  (2) FPG≥5.6 mmol/L; (3) HDL-C<1.03 mmol/L in female, <1.29 mmol/L in male, or taking antihyperlipidemic agents;  (4) fasting TG≥1.69 mmol/L or taking antihyperlipidemic agents;  (5) WC ≥90/85 cm in male/female. | BMI≥25 | MH-NW to MU-NW (25 / 326)  MH-O to MU-O (18 / 185)  MU-NW to MH-NW (6 / 123)  MU-O to MH-O (3 / 62)  MH-NW to MH-O (2 / 25)  MU-NW to MU-O (3 / 31)  MH-O to MH-NW (4 / 95)  MU-O to MU-NW (12 / 74)  MH-NW to MU-O (3 / 34)  MU-O to MH-NW (2 / 35) | During early phase of FU |
| Min 2021 | REACTION | China | 4604 | 3.0 | 258 T2DM | MH: 0-2 factors  MU: ≥3 factors  (1) BP≥130/85 mmHg or diagnosed hypertension and on antihypertensive therapy;  (2) FPG ≥6.1 mmol/L or OGTT 2hPG ≥7.8 mmol/L, or confirmed diabetes that was under treatment;  (3) fasting HDL-C <1.04 mmol/L. (4) fasting TG ≥ 1.70 mmol/ L;  (5) WC≥90/85 cm for male/female. | BMI≥24 | MH-O to MU-O (24 / 254)  MU-O to MH-O (8 / 197) | Throughout FU |
| Navarro-González 2016 | VMCUN | Europe | 4340 | 9.0 (male)  9.2 (female) | 262 T2DM | MH: 0-2 factors  MU: ≥3 factors (1) BP≥ 130/85 mm Hg or taking a pharmacological treatment for hypertension;  (2) FPG≥100 mg/dL;  (3) HDL-C <40 mg/dL in male and <50 mg/dL in female;  (4) TG ≥150 mg/dL. | BMI≥30 | MH-O to MU-O (18 / 144)  MH-O to MH-NW (2 / 59) | During early phase of FU |
| Song 2022 | - | China | 14894 | 10.1 | 9033 T2DM | MH: 0-1 factors  MU: ≥2 factors  (3) systolic BP≥130 mmHg or diastolic BP ≥85 mmHg or self-reported hypertension or anti-hypertensive drugs;  (2) fasting glucose>5.6mmol/L and <7mmol/L;  (3) HDL-C <1 mmol/L in male, <1.3mmol/L in female;  (4) triglycerides≥1.7mmol/L or lipid-lowering drugs;  (5) WC ≥90/85 cm in male/female. | BMI≥24 | MH-O to MU-O (84 / 2209)  MH-NW to MH-O (12 / 1490) | Throughout FU |
| Wang 2018 | - | China | 11865 | 6.0 | 693 T2DM | MH: 0-1 factors  MU: ≥2 factors  (1) systolic BP≥130 mmHg or diastolic BP≥85 mmHg or current treatment for hypertension;  (2) FPG ≥5.60 mmol/L.  (3) HDL-C<1.03 mmol/L in male, <1.29 mmol/L in female;  (4) fasting TG≥1.7 mmol/L. | BMI≥24 | MH-NW to MU-NW (27 / 671)  MH-O to MU-O (58 / 876)  MU-NW to MH-NW (6 / 433)  MU-O-MH-O (34 / 606)  MH-NW to MH-O (5 / 521)  MU-NW to MU-O (26 / 437)  MH-O to MH-NW (0 / 173)  MU-O to MU-NW (34 / 263)  MH-NW to MU-O (19 / 344)  MU-O to MH-NW (1 / 95) | Throughout FU |
| Cho 2019 | NHIS-HEALS | South Korea | 362863 | 2.0 | 11055 CVD events  11532 CVD-specific mortality  1942 all-cause mortality | MH: 0-1 factors  MU: ≥2 factors  (1) systolic BP≥130 mmHg and/or diastolic BP≥85 mmHg and/or taking antihypertensive treatment;  (2) FPG≥100 mg/dl and/or taking antidiabetic medications;  (3) HDL-C ≥40 mg/dl in male and ≥50 mg/dl in female; (4) TG≥150 mg/dl and/or taking antidyslipidemic medications. | BMI≥25 | MH-NW to MU-NW (436 / 25531)  MH-O to MU-O (180 / 11468)  MU-NW to MH-NW (345 / 22236)  MU-O to MH-O (160 / 9549)  MH-NW to MH-O (46 / 3768)  MU-NW to MU-O (184 / 7912)  MH-O to MH-NW (50 / 3642)  MU-O to MU-NW (238 / 9494)  MH-NW to MU-O (52 / 2874)  MU-O to MH-NW (43 / 2573) | Prior to FU |
| Eckel 2018 | NHS | US | 90257 | 24.0 | 6306 CVD events | MH: 0 factor  MU: ≥1 factors  (1) metabolic disorders hypertension;  (2) diabetes;  (3) hypercholestrolaemia. | BMI≥25 | MH-NW to MU-NW (1048 / 2965*)  MH-O to MU-O (792 / 165845*) | Prior to FU |
| Gao 2020 | China Kadoorie Biobank | China | 458246 | 10.0 | 52251 CVD events | MH: 0-2 factors  MU: ≥3 factors (1) systolic BP≥130 mmHg or diastolic BP≥85 mmHg or self-reported hypertension or using antihypertensive drugs;  (2) FPG ≥5.6 mmol/L or RPG≥ 11.1 mmol/L or self-reported diabetes; (3) reduced plasma HDL-C (<1.0 mmol/L for male and <1.3 mmol/L for female) or using lipid-lowing drugs; (4) elevated plasma TG≥1.7 mmol/L) or using lipid-lowing drugs; (5) WC≥90/85 cm in male/female. | BMI≥24 | MH-O to MU-O (453 / 6667)  MH-NW to MH-O (97 / 1498) | During early phase of FU |
| Guo 2021 | NCRCHS | China | 7472 | 4.7 | 344 CVD events | MH: 0-2 criteria MU: ≥3 criteria (1) BP: ≥130/85mmHg or use of antihypertensive drugs;  (2) serum glucose: ≥5.6mmol/L or current use of antihyperglycemic agents; (3) serum HDL-C: <1.0mmol/L in male, <1.3mmol/L in female (4) serum TGs ≥1.7mmol/L; (5) WC: ≥85/80 cm in male/female. | BMI≥25 | MH-O to MU-O (NA) | During early phase of FU |
| Hosseinpanah 2020 | TLGS | Iran | 6758 | 15.9 | 828 CVD events | MH: 0-2 factors  MU: ≥3 factors (1) systolic BP<130 mmHg, diastolic BP<85 mmHg or antihypertensive drug treatment;  (2) FPG<100 mg/dL (5.6 mmol/L) or 2-h blood glucose<140 mg/dl (7.8 mmol/L) or drug treatment;  (3) fasting HDL-C <50 mg/dL (1.29 mmol/L) in female and <40 mg/dL (1.03 mmol/L) in male or drug treatment;  (4) fasting TG<150 mg/dL (1.7 mmol/L) or drug treatment;  (5) WC<91/89 cm in male/female. | BMI≥25 | MH-O to MU-O (53 / 503, male)  (32 / 893, female) | Throughout FU |
| Lee 2022 | ASAS (KoGES) | South Korea | 6665 | 17.4 | 664 CVD events | MH: 0 factor  MU: ≥1 factors (1) systolic BP ≥140 mmHg, diastolic BP≥90 mmHg, or a self-report of taking antihypertensive mediations or diagnosis of hypertension;  (2) fasting glucose ≥126 mg/dL, self-reported use of anti-diabetic medication, or a self-reported physician diagnosis of diabetes mellitus;  (3) a total cholesterol of ≥240 mg/dL, self-reported antihyperlipidemic medication use, or a self-reported medterol. | BMI≥25 | MH-NW to MU-NW (55 / 7060*)  MH-O to MU-O (51 / 5191*)  MU-NW to MH-NW (17 / 2360*)  MU-O to MH-O (17 / 1669*)  MH-NW to MH-O (11 / 1697*)  MU-NW to MU-O (15 / 1163*)  MH-O to MH-NW (12 / 2175*)  MU-O to MU-NW (26 / 2508*)  MH-NW to MU-O (11 / 1084*)  MU-O to MH-NW (4 / 565*) | During early phase of FU |
| Lee 2020 | KNHIS | South Korea | 7148763 | 3.7 | 3151 CVD events | MH: 0-2 factors  MU: ≥3 factors (1) systolic BP ≥130 mmHg or diastolic BP ≥85 mmHg or BP medication use;  (2) fasting glucose ≥100 mg/dl; (3) HDL-C <40 mg/dl in male and <50 mg/dl in female or medication use; (4) triglyceride level ≥150 mg/dl or medication use; (5) WC ≥90/85 cm in male/female. | BMI≥25 | MH-NW to MU-NW (591 / 546422)  MH-O to MU-O (354 / 533237)  MH-NW to MH-O (120 / 420879)  MH-O to MH-NW (99 / 252858)  MH-NW to MU-O (105 / 167760) | Prior to FU |
| Lin 2020 | - | China | 6220 | 4.4 | 842 CVD events | MH: 0-1 factors  MU: ≥2 factors  (1) systolic BP ≥130 mmHg and/or diastolic BP ≥85 mmHg, or on antihypertensive treatment;  (2) FPG≥5.6 mmol/L, or on medications for diabetes; (3) triacylglycerols ≥1.7 mmol/L, or on lipid-lowering medications; (4) HDL-C <1.04 mmol/L in male and <1.29 mmol/L in female. | BMI≥25 | MH-NW to MU-NW (27 / 675)  MH-O to MU-O (21 / 515)  MU-NW to MH-NW (2 / 262)  MU-O to MH-O (6 / 292) | Throughout FU |
| Mongraw-Chaffin 2018 | MESA | US | 5005 | 12.2 | 791 CVD events  975 all-cause mortality | MH: 0-2 factors  MU: ≥3 factors  (1) systolic BP ≥130mmHg and/or diastolic BP≥85mmHg;  (2) fasting glucose ≥100 mg/dL;  (3) HDL-C<40mg/L in male, <50mg/L in female; (4) triglycerides: ≥150mg/L; (5) WC: ≥102/88 cm in male/female. | BMI≥30 | MH-O to MU-O (51 / 501) | Throughout FU |
| Mørkedal 2014 | HUNT | Norway | 61299 | 12.2 | 2547 CVD events | MH: 0-1 factors  MU: ≥2 factors  (1) BP≥130/85 mmHg or use of BP medication;  (2) nonfasting glucose ≥11.1 mmol/L, or diabetes diagnosis; (3) HDL-C <1.03 mmol/L in male, <1.29 mmol/L in female;  (4) nonfasting triglycerides ≥1.7 mmol/L; | BMI≥25 | MH-NW to MH-O (16 / NA)  MU-NW to MU-O (70 / NA) | Prior to FU |

MH: metabolically healthy; MU: metabolically unhealthy; MH-NW: metabolically healthy normal weight; MH-O: metabolically healthy overweight/obesity; MU-NW: metabolically unhealthy normal weight; MU-O: metabolically unhealthy overweight/obesity; T2DM: type 2 diabetes mellitus; CVD: cardiovascular disease; BP: blood pressure; FPG: fasting plasma glucose; RPG: random plasma glucose; HLD-C: high-density lipoprotein-cholesterol; WC: waist circumference; * events / person-years.

**Additional file 1: Table S3.** Results of quality assessment (n=19).

| Study ID | Outcome | Selection | | | |  | Comparability |  | Outcome | | | Total stars | Overall quality |  |
| --- | --- | --- | --- | --- | --- | --- | --- | --- | --- | --- | --- | --- | --- | --- |
|  |  | Representative-  ness of the exposed cohort | Selection of the non-exposed cohort | Ascertainment of exposure | Demonstration that outcome of interest was not present at start of study |  | Comparability of cohorts on the basis of the design or analysis controlled for confounders |  | Ascertainment of outcome | Follow-up long enough for outcomes to occur | Adequacy of follow-up of cohorts |  |  |  |
| Feng 2020 | T2DM | NA | * | * | * |  | ** |  | * |  | * | 7 | High |  |
| Heianza 2014 | T2DM | NA | * | * | * |  | ** |  | * | * |  | 7 | High |  |
| Lee 2015 | T2DM | NA | * | * | * |  | ** |  | * | * |  | 7 | High |  |
| Min 2021 | T2DM | NA | * | * | * |  | ** |  | * |  |  | 6 | Moderate |  |
| Navarro-González 2016 | T2DM | NA | * | * | * |  | ** |  | * | * |  | 7 | High |  |
| Song 2022 | T2DM | NA | * |  | * |  | ** |  | * | * |  | 6 | Moderate |  |
| Wang 2018 | T2DM | NA | * |  | * |  | ** |  | * | * | * | 7 | High |  |
| Cho 2019 | CVD events | NA | * | * | * |  | ** |  | * |  | * | 7 | High |  |
| Eckel 2018 | CVD events | NA | * |  | * |  | ** |  | * | * | * | 7 | High |  |
| Gao 2020 | CVD events | NA | * | * | * |  | ** |  | * | * | * | 8 | High |  |
| Guo 2021 | CVD events | NA | * | * | * |  | ** |  | * | * | * | 8 | High |  |
| Hosseinpanah 2020 | CVD events | NA | * | * | * |  | ** |  | * | * | * | 8 | High |  |
| Lee 2022 | CVD events | NA | * |  | * |  | ** |  |  | * | * | 6 | Moderate |  |
| Lee 2020 | CVD events | NA | * | * | * |  | ** |  | * | * |  | 7 | High |  |
| Lin 2020 | CVD events | NA | * | * | * |  | ** |  | * |  |  | 6 | Moderate |  |
| Mongraw-Chaffin 2018 | CVD events | NA | * |  | * |  | ** |  |  | * |  | 5 | Moderate |  |
| Mørkedal 2014 | CVD events | NA | * | * | * |  | ** |  | * | * |  | 7 | High |  |

T2DM: type 2 diabetes mellitus; CVD: cardiovascular disease; NA: not applicable.

**Figure S1.** Results of subgroup analysis for MH-O to MU-O and risk for T2DM

**Figure S2.** Results of subgroup analysis for transition from MH-O to MU-O and risk of composite CVD events

**Figure S3.** The forest plots for risk of CVD outcomes as originally reported in studies and all-cause mortality

(A)

(B)

**Figure S4.** Funnel plots for transition from MH-O to MU-O and risk of CVD events: (A) original funnel plot (Egger’s test p=0.04, pooled HR [1.46, 95% CI 1.32, 1.62]); (B) funnel plots generated from trim-and-fill method (pooled HR [1.42, 95% CI 1.28, 1.57]).

**Additional file 1: Table S4.** Certainty of evidence assessed by GRADE for associations with risk of T2DM

| No. of  Cohorts | Study Design | Risk of Bias | Inconsistency | Indirectness | Imprecision | Publication Bias | Other | Study Event  Rates (%) | Hazard Ratio  (95% CI) | Certainty |
| --- | --- | --- | --- | --- | --- | --- | --- | --- | --- | --- |
| MH-NW to MU-NW and risk of T2DM | | | | | | | | | | |
| 4 | Observational | Not serious | Not serious | Not serious | Not serious | Undetected ^1^ | Lage effect size ^2^ | 3,205/91,737  (3.5%) | 2.87 (2.43, 3.38) | ⊕⊕⊕○ Moderate |
| MH-O to MU-O and risk of T2DM | | | | | | | | | | |
| 8 | Observational | Not serious | Not serious | Not serious | Not serious | Undetected ^1^ | Lage effect size ^2^ | 12,758/115,575 (11.0%) | 2.68 (2.07, 3.47) | ⊕⊕⊕○ Moderate |
| MU-NW to MH-NW and risk of T2DM | | | | | | | | | | |
| 3 | Observational | Not serious | Not serious | Not serious | Not serious | Undetected ^1^ | Lage effect size ^2^ | 1,950/64,259 (3.0%) | 0.40 (0.29, 0.55) | ⊕⊕⊕○ Moderate |
| MU-O to MH-O and risk of T2DM | | | | | | | | | | |
| 4 | Observational | Not serious | Not serious | Not serious | Not serious | Undetected ^1^ | Lage effect size ^2^ | 2,420/46,639 (5.2%) | 0.34 (0.18, 0.64) | ⊕⊕⊕○ Moderate |
| MH-NW to MH-O and risk of T2DM | | | | | | | | | | |
| 3 | Observational | Not serious | Not serious | Not serious | Serious ^3^ | Undetected ^1^ | None | 11,195/56,929 (19.7%) | 1.30 (0.79, 2.12) | ⊕○○○  Very low |
| MU-NW to MU-O and risk of T2DM | | | | | | | | | | |
| 3 | Observational | Not serious | Not serious | Not serious | Serious ^4^ | Undetected ^1^ | None | 2,162/42,035 (5.1%) | 1.31 (0.92, 1.88) | ⊕○○○  Very low |
| MH-O to MH-NW and risk of T2DM | | | | | | | | | | |
| 3 | Observational | Not serious | Serious ^5^ | Not serious | Not serious | Undetected ^1^ | Lage effect size ^2^ | 1,519/34,510 (4.4%) | 0.41 (0.21, 0.78) | ⊕⊕○○  Low |
| MU-O to MU-NW and risk of T2DM | | | | | | | | | | |
| 3 | Observational | Not serious | Not serious | Not serious | Not serious | Undetected ^1^ | None | 2,162/42,035 (5.1%) | 0.71 (0.55, 0.91) | ⊕⊕○○  Low |
| MH-NW to MU-O and risk of T2DM | | | | | | | | | | |
| 3 | Observational | Not serious | Not serious | Not serious | Not serious | Undetected ^1^ | Lage effect size ^2^ | 2,162/42,035 (5.1%) | 4.76 (3.12, 7.26) | ⊕⊕⊕○ Moderate |
| MU-O to MH-NW and risk of T2DM | | | | | | | | | | |
| 3 | Observational | Not serious | Not serious | Not serious | Not serious | Undetected ^1^ | Lage effect size ^2^ | 2,162/42,035 (5.1%) | 0.24 (0.11, 0.51) | ⊕⊕⊕○ Moderate |

^1^ No downgrade for publication bias as publication bias could not be assessed due to lack of power for assessing funnel plot asymmetry and small study effects (i.e. <10 observations available).

^2^ Upgrade for large size effect (HR<0.5 or >2).

^3^ Downgrade because the conﬁdence interval included a reduction (21% reduction) and a substantial increase (112% increase).

^4^ Downgrade because the conﬁdence interval included a small reduction (8% reduction) and a substantial increase (88% increase).

^5^ Downgrade for serious inconsistency given evidence of substantial inter-study heterogeneity (I^2^=93.3%, p=0.02), which could not be explored through subgroup analysis due to only 3 observations available.

**Additional file 1: Table S5.** Certainty of evidence assessed by GRADE for associations with risk of CVD

| No. of  Cohorts | Study Design | Risk of Bias | Inconsistency | Indirectness | Imprecision | Publication Bias | Other | Study Event Rates (%) | Hazard Ratio  (95% CI) | Certainty |
| --- | --- | --- | --- | --- | --- | --- | --- | --- | --- | --- |
| MH-NW to MU-NW and risk of CVD | | | | | | | | | | |
| 5 | Observational | Not serious | Not serious | Not serious | Not serious | Undetected ^1^ | None | 22,018/7,614,768 (0.3%) | 1.40 (1.31, 1.49) | ⊕⊕○○  Low |
| MH-O to MU-O and risk of CVD | | | | | | | | | | |
| 11 | Observational | Not serious | Serious ^2^ | Not serious | Not serious | Not serious ^3^ | None | 76,232/8,092,249 (0.9%) | 1.46 (1.32, 1.62) | ⊕○○○  Very low |
| MU-NW to MH-NW and risk of CVD | | | | | | | | | | |
| 3 | Observational | Not serious | Not serious | Not serious | Not serious | Undetected ^1^ | None | 12,561/375,748 (3.3%) | 0.71 (0.63, 0.80) | ⊕⊕○○  Low |
| MU-O to MH-O and risk of CVD | | | | | | | | | | |
| 3 | Observational | Not serious | Not serious | Not serious | Serious ^4^ | Undetected ^1^ | None | 12,561/375,748 (3.3%) | 0.87 (0.74, 1.02) | ⊕○○○  Very low |
| MH-NW to MH-O and risk of CVD | | | | | | | | | | |
| 6 | Observational | Not serious | Not serious | Not serious | Not serious | Undetected ^1^ | None | 69,668/8,037,836 (0.9%) | 1.18 (1.05, 1.32) | ⊕⊕○○  Low |
| MU-NW to MU-O and risk of CVD | | | | | | | | | | |
| 4 | Observational | Not serious | Not serious | Not serious | Serious ^5^ | Undetected ^1^ | None | 14,266/430,827 (3.3%) | 1.14 (0.87, 1.48) | ⊕○○○  Very low |
| MH-O to MH-NW and risk of CVD | | | | | | | | | | |
| 3 | Observational | Not serious | Serious ^6^ | Not serious | Serious ^7^ | Undetected ^1^ | None | 14,870/7,518,291 (0.2%) | 0.81 (0.54, 1.22) | ⊕○○○  Very low |
| MU-O to MU-NW and risk of CVD | | | | | | | | | | |
| 2 | Observational | Not serious | Serious ^8^ | Not serious | Serious ^9^ | Undetected ^1^ | None | 11,719/369,528 (3.2%) | 0.83 (0.53, 1.31) | ⊕○○○  Very low |
| MH-NW to MU-O and risk of CVD | | | | | | | | | | |
| 3 | Observational | Not serious | Not serious | Not serious | Not serious | Undetected ^1^ | None | 14,870/7,518,291 (0.2%) | 1.44 (1.18, 1.74) | ⊕⊕○○  Low |
| MU-O to MH-NW and risk of CVD | | | | | | | | | | |
| 2 | Observational | Not serious | Not serious | Not serious | Serious ^10^ | Undetected ^1^ | None | 11,719/369,528 (3.2%) | 0.77 (0.57, 1.03) | ⊕○○○  Very low |

^1^ No downgrade for publication bias as publication bias could not be assessed due to lack of power for assessing funnel plot asymmetry and small study effects (i.e. <10 observations available).

^2^ Downgrade for serious inconsistency given the evidence of substantial inter-study heterogeneity (I^2^=79.9%, p=0.02).

^3^ No downgrade for publication bias as the validity of result was confirmed by trim-and-fill methods despite evidence of publication bias detected by Egger’s test (p<0.10).

^4^ Downward as the conﬁdence interval included a substantial reduction (26% reduction) and a small increase (2% increase).

^5^ Downward as the conﬁdence interval included a small reduction (13% reduction) and a substantial increase (48% increase).

^6^ Downgrade for serious inconsistency given evidence of substantial inter-study heterogeneity (I^2^=93.3%, p=0.03), which could not be explored through subgroup analysis due to only 3 observations available.

^7^ Downward as the conﬁdence interval included a substantial reduction (46% reduction) and an increase (22% increase).

^8^ Downgrade for serious inconsistency given evidence of substantial inter-study heterogeneity (I^2^=95.3%, p=0.04), which could not be explored through subgroup analysis due to only 2 observations available.

^9^ Downward as the conﬁdence interval included a substantial reduction (47% reduction) and an increase (31% increase).

^10^ Downward as the conﬁdence interval included a substantial reduction (43% reduction) and a small increase (3% increase).
